# Therapeutic effect of Bacillus Calmette–Guerin polysaccharide nucleic acid on mast cell at the transcriptional level

**DOI:** 10.7717/peerj.7404

**Published:** 2019-08-21

**Authors:** Siyu Yan, Runqiu Liu, Manyun Mao, Zhaoqian Liu, Wei Zhang, Yi Zhang, Jie Li, Cong Peng, Xiang Chen

**Affiliations:** 1 Department of Dermatology, Xiangya Hospital of Central South University, Changsha, Hunan, China; 2 Hunan Engineering Research Center of Skin Health and Disease, Xiangya Hospital of Central South University, Changsha, Hunan, China; 3 Hunan Key Laboratory of Skin cancer and Psoriasis, Xiangya Hospital of Central South University, Changsha, Hunan, China; 4 Institute of Clinical Pharmacology, Xiangya Hospital, Changsha, China; 5 JIUZHITANG Medicine Commerce CO, LTD, Changsha, China

**Keywords:** BCG-PSN, Chronic spontaneous urticaria, Combined therapeutic effect, Mast cell

## Abstract

**Background:**

Chronic spontaneous urticaria (CSU) is a common and recurrent autoimmune-related disease with unclear pathogenesis. Dysfunction of immune cells, such as T cells, mast cells, and basophils, is involved. Bacillus Calmette–Guerin polysaccharide nucleic acid (BCG–PSN), an immunomodulator partially extracted from BCG, can be used in the combined treatment of CSU with an unknown mechanism.

**Methods:**

To study the therapeutic effect and mechanism of BCG–PSN on CSU, we initially assessed the clinical efficacy in 110 enrolled CSU patients of 4-week antihistamine monotherapy vs. antihistamine plus BCG–PSN combined therapy. Subsequently, to explore the further mechanism of BCG-PSN, the mast cell line RBL-2H3 pretreated with BCG-PSN was used to evaluate the transcriptional expression profiles via lncRNA sequencing. Real time PCR was conducted to validate the candidate gene expression.

**Results:**

We found no significant difference in treatment efficacy between the BCG–PSN group (71.7%) and the monotherapy group (71.9%). However, the average time of complete relief in the BCG–PSN group was significantly shorter than that in the monotherapy group (36.77 ± 17.33 vs. 51.27 ± 16.80, *p* = 0.026). *In vitro* experiments showed that BCG-PSN inhibited β-hexosaminidase release rates in IgE-sensitized RBL-2H3 cells (*p* < 0.001). Sequencing data revealed the expression profiles of functional genes, including a significant decrease in Erb-B2 receptor tyrosine kinase 4, which can be regulated by the nuclear factor kappa B (NF-κB) pathway.

**Discussion:**

CSU is a chronic, recurrent disease with complex pathogenesis. Mast cells and basophils are the primary target cells of the disease. BCG–PSN decrease the β-HEX release rates and regulated IgE-mediated mast cell activation in RBL-2H3 cells by mediating immune-related gene expression including ERBB4. These findings suggest that BCG–PSN may mediate ERBB4 expression *via* the NF-κB pathway and may have value in the treatment of CSU.

## Introduction

Chronic urticaria, a common and recurrent disease partly associated with autoimmunity (Altman & Chang, 2013; Confino-Cohen et al., 2012; Di Lorenzo et al., 2013; Zuberbier et al., 2018), is defined as having wheals, pruritus, and/or angioedema for more than six weeks. Chronic spontaneous urticaria (CSU) is the most common subtype of this disease (Zhong et al., 2014). According to previous studies, CSU negatively impacted patients’ health-related quality of life and was associated with high medical costs due to recurrent and persistent wheals and pruritus (Choi et al., 2016; Delong et al., 2008; Maurer, Ortonne & Zuberbier, 2009). The severity of the impact of its symptoms on quality of life is like that of chronic ischemic heart disease (Maurer et al., 2013). A recent cohort study of 673 adults with CSU reported a higher economic and humanistic burden compared with general matched controls, and more severe patients were affected to a greater extent (Balp et al., 2017; Maurer et al., 2017). Approximately 35%–40% of patients with CSU have an autoimmune basis (Ferrer, 2015). Mast cells or basophils play significant roles in the pathogenesis of CSU; cross-linking of immunoglobulin E (IgE) and the Fc fragment of the IgE receptor Ia participate in the activation of mast cells (Bonnekoh et al., 2018). T cells are also known to be engaged in this process (Jain, 2014; Kolkhir et al., 2017b). Compared with healthy controls, CSU patients present abnormal Th1/Th2 cytokine levels, including increased IL-6 and decreased IFN-γ (Kasperska-Zajac, Grzanka & Damasiewicz-Bodzek, 2015; Lopes et al., 2013; Moy, Murali & Nazarian, 2016).

Nonsedating antihistamines are considered the first-line treatment for CSU based on the newest EAACI/GA^2^LEN/EDF/WAO guidelines (Zuberbier et al., 2018). If the previous treatment is not effective, then increasing the dose seems to be necessary. When responding poorly after 2–4 weeks of further therapy, immunomodulators such as cyclosporin can be used to relieve symptoms accompanied by antihistamines (Ferrer et al., 2015; Powell et al., 2015). Antihistamines and immunomodulators stabilize mast cells, inhibit Th2 cytokine release, and attenuate leukotriene production (Bunikowski et al., 2001; Ortonne, 2012; Plath, Grabbe & Gibbs, 2003).

Immunomodulators such as Bacillus Calmette–Guerin polysaccharide nucleic acid (BCG–PSN) participate in immunomodulatory actions (Li et al., 2013). BCG–PSN is a mixture of nucleic acids and polysaccharides extracted from BCG immune-active substances. It has been previously used in allergic diseases, including asthma, atopic dermatitis, and chronic urticaria (Li et al., 2013; Sun et al., 2013). BCG–PSN promotes the proliferation and activation of T cells and stimulates mononuclear cells by affecting the synthesis and secretion of cytokines, such as IFN-γ (Hu & Chen, 2002; Li et al., 2004). It also activates TLR signaling by increasing Th1-type cytokine levels (Sun et al., 2013). Patients treated with BCG–PSN were demonstrated to have increased IL-2 and decreased IL-10 levels in their peripheral blood mononuclear cells (Li et al., 2013).

Previous studies on BCG–PSN focused on the regulation of T cells and paid little attention to mast cells or basophils. RBL-2H3 cells are commonly used to explore mast cell function *in vitro* (Passante & Frankish, 2009). In this study, we aimed to investigate the therapeutic efficacy of BCG-PSN combined with antihistamines in CSU patients and explore the underlying mechanism of its impact on gene expression at the transcriptional level in RBL-2H3 cells.

## Materials and Methods

### Patient enrollment

Patients with CSU who were treated with nonsedating H1 antihistamine with or without BCG–PSN during September 2013–December 2013 from Xiangya Hospital were recruited. This cross-sectional study was approved by the Ethics Committee of Xiangya Hospital (201311392). All patients enrolled in our study signed the written informed consent. Each patient was treated with antihistamine monotherapy using conventional doses including desloratadine 5 mg/d and levocetirizine 5 mg/d, with or without BCG–PSN. The enrollment of CSU patients was according to a previous study (Yan et al., 2014; Guo et al., 2015), while patients with severe allergic symptoms, acute disorders, infectious diseases, or glucocorticoid treatment were excluded. The disease severity was assessed by weekly urticaria activity score (UAS7), and when the UAS7 score decreased to 0, the patient was considered as complete relief.

### Cell culture

RBL-2H3 cells (ATCC, Manassas, VA, USA) were cultured in 1640 medium (Biological Industries, Israel) with 15% fetal bovine serum, 100 U/ml penicillin, and 100 mg/ml streptomycin at 37 °C with 5% CO_2_ in a humidified atmosphere.

### β-Hexosaminidase (β-HEX) assay

RBL-2H3 cells at 90% confluence were inoculated into a six-well plate at a density of 2 × 10^5^ cells/ml. After overnight sensitization by anti-DNP-IgE (100 ng/ml), cells were pretreated with different concentrations (0, 20, 40, 80, or 100 µl/ml) of BCG–PSN (Jiuzhitang Co., Ltd) at different time points (1, 2, 4, or 6 h) with mizolastine or mizolastine alone. After washing with Tyrode’s buffer twice, the cells were incubated with DNP–HSA (1 µg/ml, Alpha Diagnostic Inc.) for 30 min at 37 °C. The supernatant of each cell culture was withdrawn, and cells were lysed with NP-40 (3 mM) of the same volume. β-HEX solution (50 µl, 3 mM *N*-acetyl-β-D-glucosaminide, Sigma) was added to 50 µl of the supernatant, which was then mixed and incubated for 90 min at 37 °C. Subsequently, the reaction was suspended by the addition of 150 µl NaHCO_3_/Na_2_CO_3_ solution (pH = 10.5), and the absorbance of the supernatant was detected at 405 nm. The β-HEX release rate was evaluated as described previously (Li et al., 2017).

### Cell viability assay

RBL-2H3 cells were seeded into a 96-well culture plate at a cell density of 4000 cells/well. Different concentrations (0, 20, 40, 80, or 100 µl/ml) of BCG–PSN were added to each well. At estimated time points, PMS solution premixed with MTS (1:40) was added into each well and reacted for 2 h at 37 °C, and the absorbance of the supernatant was evaluated at 450 nm. The experiments were conducted at least in triplicate, and readings were normalized to cells treated with control.

### LncRNA sequencing

RBL-2H3 cells were seeded into a six-well plate and sensitized with anti-DNP-IgE (100 ng/ml) overnight , then mixed with the appropriate concentration of BCG–PSN for 1 h. After the incubation, DNP-HSA (one µg/ml) was used to stimulate cells for 30 min. Cells were collected subsequently, and total RNA was extracted according to the manufacturer’s instructions. LncRNA sequencing was conducted with an Illumina HiSeq 3000 system. Differential expression was tested between the BCG–PSN drug group and the negative control using the unpaired *t*-test. LncRNAs and mRNAs with a fold change of more than 2.0 and a corrected *p*-value less than 0.05 were assessed as differentially expressed.

### Real-time PCR (RT-PCR)

RT-PCR was used to verify the differential expression of candidate genes. RNA was extracted from sensitized RBL-2H3 cells pretreated or not with the drug at the appropriate concentrations. Then, two µg of the total RNA was reverse-transcribed to cDNA via the PrimeScript™ Reverse Transcriptase kit (Takara Bio Inc.) according to the manufacturer’s instructions. RT-PCR was performed using an Applied Biosystems 7500 real-time PCR system (ABI, USA). All expression levels were presented as fold changes and were analyzed by the 2^−ΔΔCT^ method with GAPDH as the internal reference (Table S1).

### Statistical analysis

Continuous variables were presented as the mean ± standard deviation (SD). Student’s *t*-test was used to compare differences between patients receiving and not receiving BCG–PSN and the transcriptional levels of different genes. Categorical variables were expressed as count (%) and compared using the chi-square test. Data analysis was performed using SPSS 20.0 (SPSS Inc., Chicago, IL, USA). A *p*-value less than 0.05 was considered statistically significant. The significance level for multiple comparisons among genes was adjusted by Bonferroni correction.

## Results

### Efficacy of BCG–PSN in combination with antihistamines

A total of 110 patients were enrolled in this study. Among the participants, 53 were treated with BCG–PSN accompanied by antihistamine monotherapy, while 57 were treated with antihistamine monotherapy. The demographic and clinical characteristics of the patients were shown in Table 1. Age, gender, course, and baseline disease severity were not significantly different between groups. According to Table S2, the rate of treatment effectiveness after 4 weeks was not significantly different between the BCG–PSN group (71.7%) and the monotherapy group (71.9%). However, the average time of complete relief in the BCG–PSN group was significantly shorter than that in the monotherapy group (*p* = 0.026, Table 1).

**Table 1:** The clinical data of chronic spontaneous urticaria patients prescribing with different drugs. Each group data was shown as mean  ± standard deviation (SD). Categorical variable was expressed as count (percent) and compared using chi-square test.

	Antihistamines- monotherapy group	BCG-PSN combined therapy group	*P* value
Age(year)	36.63 ± 13.49	33.66 ± 12.43	0.236
Male	22(38.6%)	16(30.2%)	0.424
Female	35(61.4%)	37(69.8%)	
Duration(month)	20.23 ± 31.70	23.85 ± 33.18	0.555
Baseline UAS7	26.28 ± 9.61	24.30 ± 9.12	0.269
Time of complete relief(day)	51.27 ± 16.80	36.77 ± 17.33	0.026

**Notes.**

* *p* value less than 0.05.

### β-HEX release rate in sensitized RBL-2H3 cells pretreated with BCG–PSN

**Figure 1:**
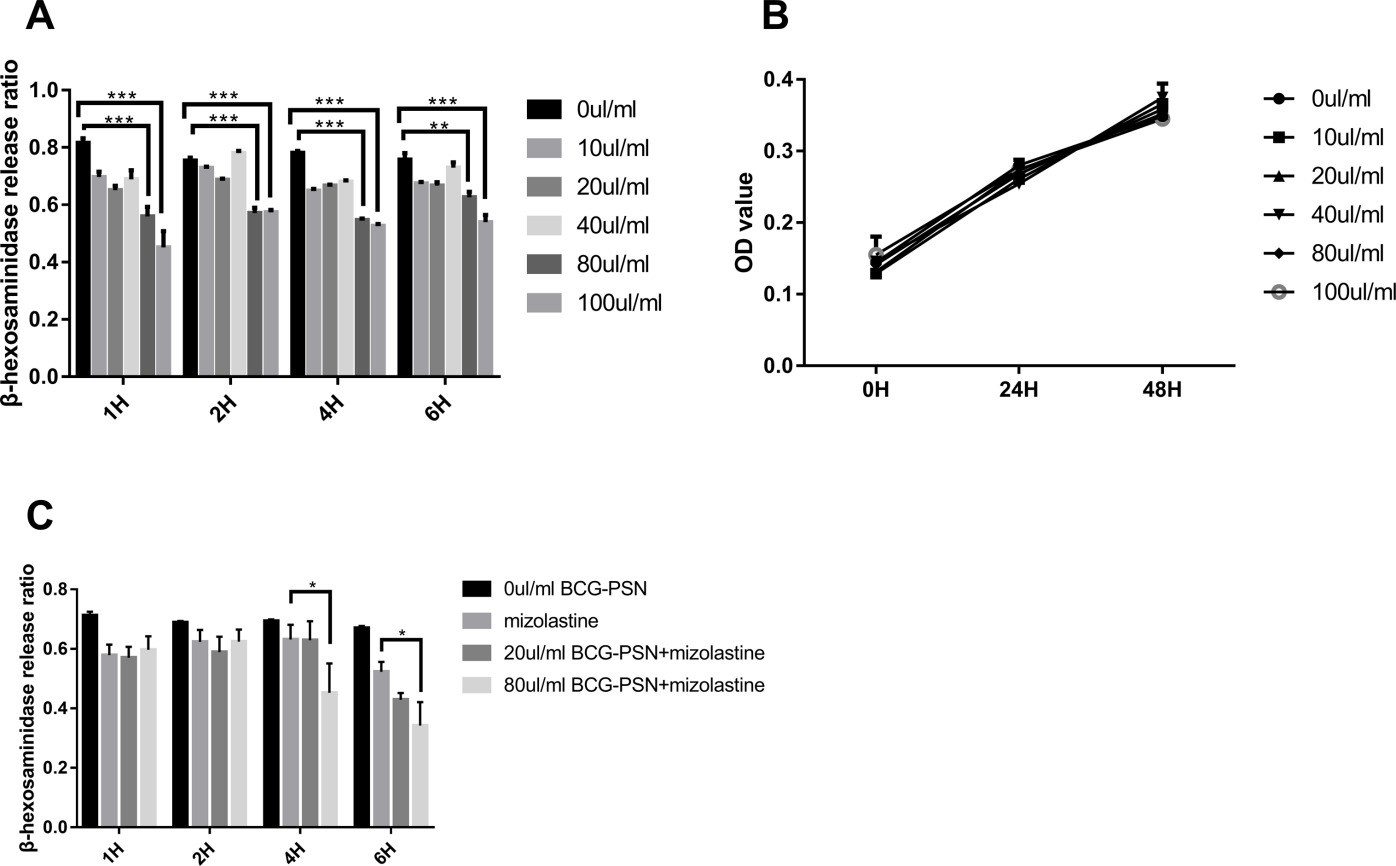
Cell viability and β-HEX release ratio at different time point on various BCG-PSN concentration in the RBL-2H3 cell. (A) is β-HEX release assay of sensentized RBL-2H3 cells treated with various BCG-PSN concentration at different time point, *n* = 3; and (B) is cell viability of RBL-2H3 cells treated with diverse BCG-PSN concentration, *n* = 5; (C) is β-HEX release assay of sensentized RBL-2H3 cells treated with mizolastine or BCG-PSN combined therapy, *n* = 3; *p-value less than 0.05, **p-value less than 0.01, ***p-value less than 0.001.

After stimulation with DNP-HSA (1 µg/ml) for 30 min, the release rate of β-HEX decreased in RBL-2H3 cells in a manner that was associated with BCG–PSN concentration. The β-HEX release ratio decreased most extensively when cells were treated with 100 µl/ml BCG–PSN (*p* < 0.001, Fig. 1A). The MTS assay showed that the cell viability was not significantly inhibited between groups treated with varying concentrations of BCG–PSN (Fig. 1B). Therefore, no more than 100 µl/ml BCG–PSN was considered an appropriate concentration for treating RBL-2H3 cells. As BCG-PSN was used for combined therapy with nonsedating antihistamines, we subsequently evaluated the mast cell activation by the BCG-PSN combined with or without the representative antihistamine mizolastine. The degranulation level of the BCG-PSN combined treatment group showed a lower β-HEX release ratio compared with either BCG-PSN or mizolastine (Fig. 1C), which indicated that BCG-PSN inhibited the sensitized mast cell activation and that this effect was enhanced when BCG-PSN was combined with the nonsedating antihistamine mizolastine.

### Expression of mRNA and lncRNA via heatmap analysis and mRNA expression validation in sensitized RBL-2H3 cells pretreated with BCG–PSN

As shown above, BCG-PSN decreased the degranulation level of sensitized RBL-2H3 cells with an unknown mechanism. Therefore, lncRNA sequencing was conducted to explore the deeper mechanism at the transcription levels, including mRNA and lncRNA expression levels. During sequencing, we first qualified the sample RNA libraries. Q30% ranged from 94.02% to 94.83%, and all libraries were appropriate for the following analysis. Detailed data are shown in Table S3.

According to the sequencing data analysis, a total of 6368 lncRNAs were detected preliminarily, and after PLEK, CNCI, and CPAT computer-filtered analysis, only 2126 lncRNAs remained. The classifications of all candidate lncRNAs are shown in Fig. S1.

**Figure 2:**
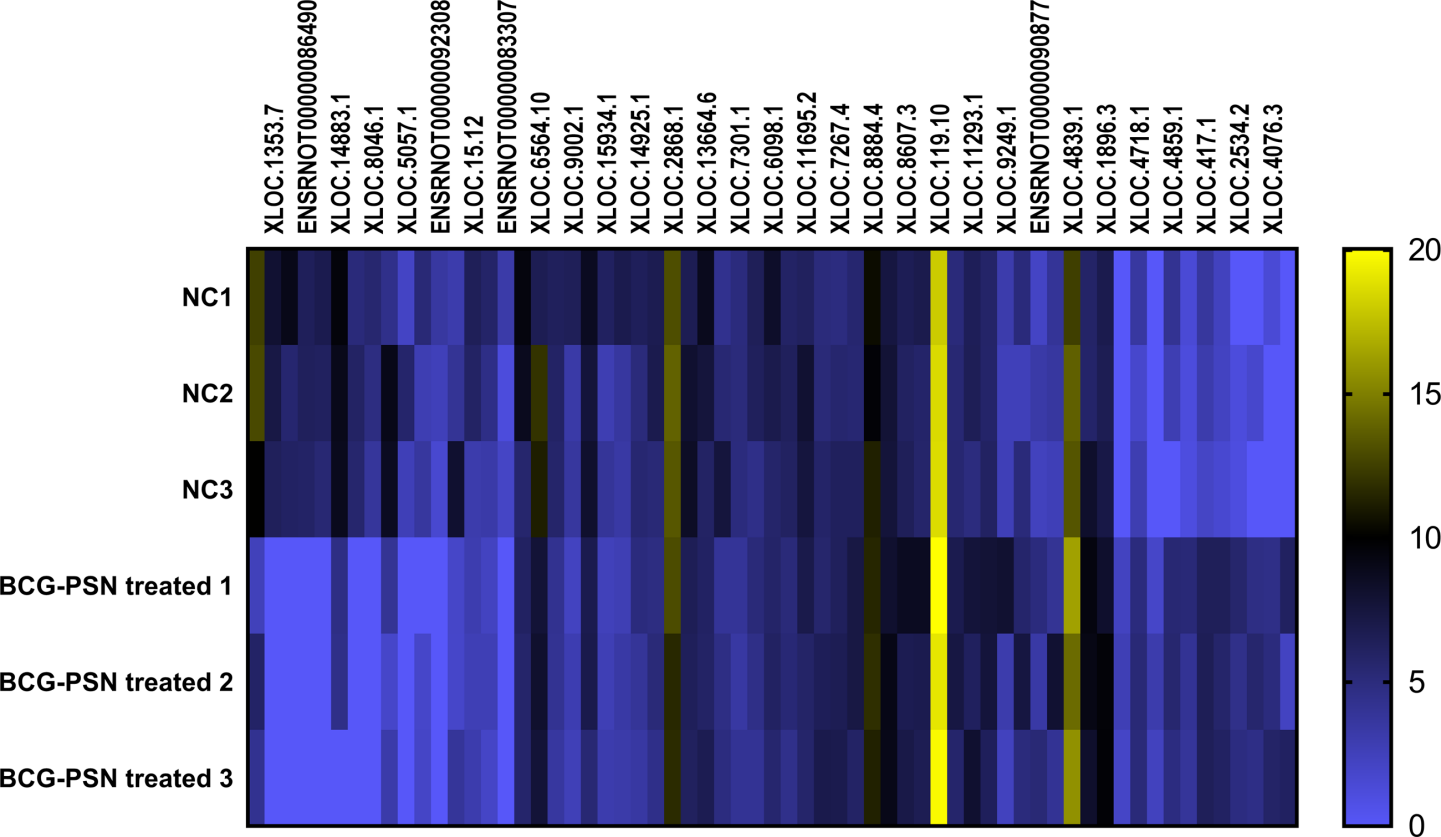
Heatmap analysis of differentially expressed lncRNA levels. Gene expression levels were depicted with different color ranging from yellow to light purple.

**Figure 3:**
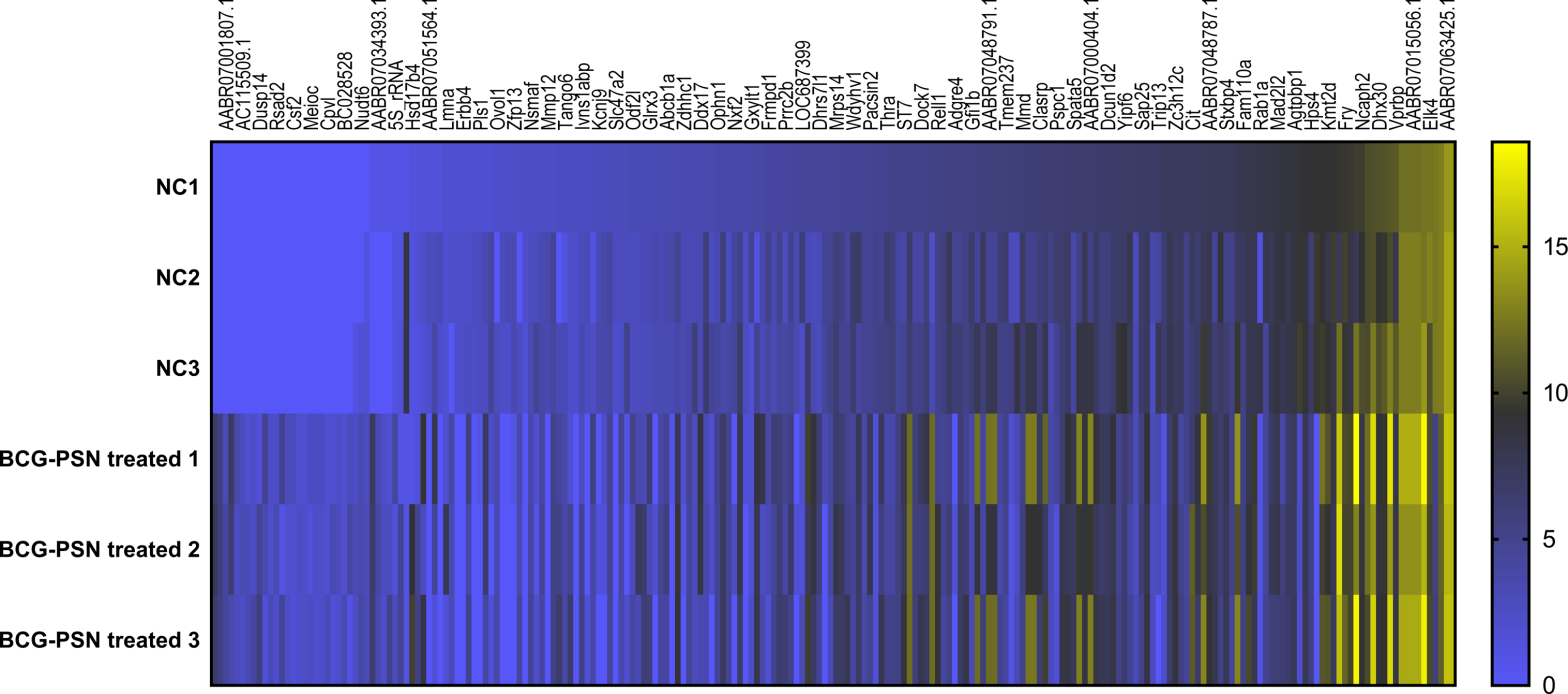
Heatmap analysis of differentially expressed mRNA levels. Gene expression levels were depicted with different color ranging from yellow to light purple. Gene symbols were illustrated on the top of the figure.

**Figure 4:**
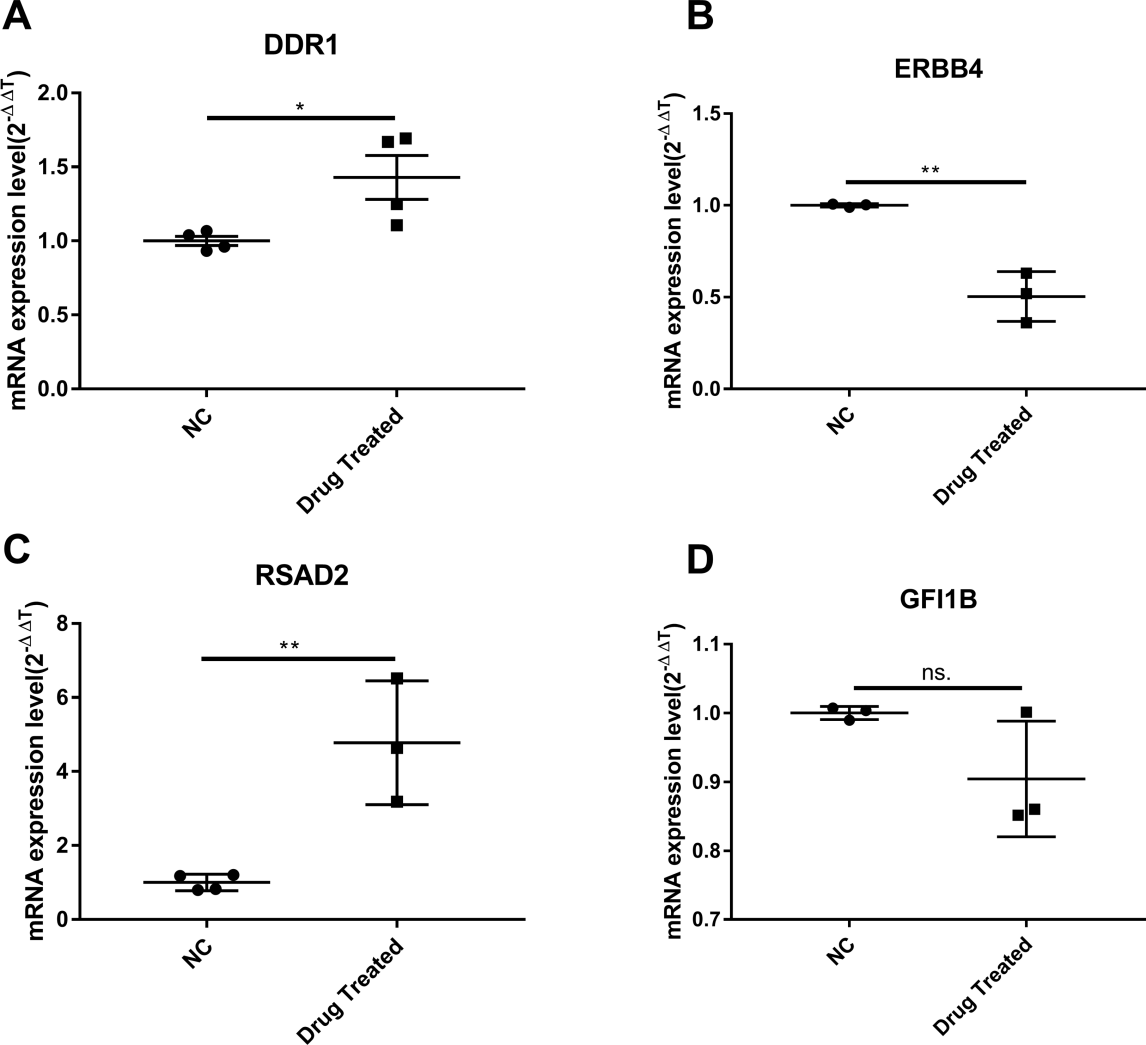
The validation of significant expression mRNAs in BCG-PSN treated RBL-2H3 cell. (A–D) represented genes including DDR1, ERBB4, RSAD2,GFI1B expression levels and were validated via RT-PCR. *p-value less than 0.05, **p-value less than 0.01.

Cluster analysis was performed using a heatmap analysis including mRNA and lncRNAs. All statistics were divided into control (IgE sensitized cells) and treatment groups (IgE sensitized cells pretreated with BCG-PSN) in triplicates. We observed a significant difference in the expression of lncRNAs (Fig. 2) and mRNAs (Fig. 3) between the groups treated with and without BCG-PSN. After setting the filter criteria of log_2_(fold change) >1 or <−1 and a *p*-value <0.05, we observed that 34 lncRNAs and 84 mRNAs were downregulated and 29 lncRNAs and 136 mRNAs were upregulated in the BCG–PSN treatment group compared with the control group (Table S4). Among them, TCR signaling and cytokine release-related genes, including growth factor independent 1B transcriptional repressor (GFI1B) and Erb-B2 receptor tyrosine kinase 4 (ERBB4), were downregulated, while a radical S-adenosyl methionine domain containing 2 (RSAD2) was upregulated (Table S5). Gene ontology (GO) analysis of the differentially expressed genes showed significant enrichment of proteins targeting intracellular organelles, membrane-bounded organelles, and intracellular parts. The upregulation of tyrosine phosphorylation pathway related genes such as Colony Stimulating Factor 2 (CSF2) and ERBB4 also showed significance. To further validate significantly differentially expressed genes, RT-PCR was performed. Validation of the mRNA levels of genes such as GFI1B, discoidin domain receptor tyrosine kinase 1 (DDR1), RSAD2, and ERBB4 showed significant downregulation of ERBB4 and upregulation of RSAD2, while the changes of DDR1 and GFI1B showed no significances (Fig. 4).

## Discussion

CSU is a chronic, recurrent disease with complex pathogenesis. Mast cells and basophils are the primary target cells of this disease (Kolkhir et al., 2017a; Kolkhir et al., 2017b). An increasing number of researchers have begun to focus on the application of high-throughput sequencing to explore the pathogenesis of complex genetic skin diseases, including psoriasis, atopic dermatitis, and systemic lupus erythematosus, at the transcriptional levels with differentially expressed genes, and even transcription factors (Flint et al., 2015; Suarez-Farinas et al., 2015; Swindell et al., 2014). Only a few studies have examined mRNA expression in CSU (Gimenez-Arnau et al., 2017; Patel et al., 2015). Researchers have observed that lesion skins expressed higher levels of inflammation-related genes than non-lesions. A recent study on CSU using a microarray also showed imbalanced immunological skin conditions with differential gene expression between lesions and non-lesions (Gimenez-Arnau et al., 2017). However, the underlying mechanism of the effect of BCG-PSN pretreatment on mast cells remains unclear. In the current study, we used the widely accepted *in vitro* model RBL-2H3 cells to study the function of mast cells pretreated with BCG-PSN (Passante & Frankish, 2009). Our research explores the mechanism of the immunomodulator BCG–PSN on rat mast cells through lncRNA sequencing for the first time.

BCG–PSN is extracted from BCG and is used for mediating immune responses in asthma, vitiligo, lichen planus, and chronic urticaria (Mou & Zheng, 2015; Jin et al., 2009; Xiong et al., 2009; Zhan, Xiong & Wang, 2014). A clinical survey on antihistamine therapy with or without BCG–PSN showed beneficial effects of BCG-PSN during CSU treatment without apparent toxicity (Wang, 2013). In our study, we observed no significant difference regarding efficacy between the groups with and without BCG–PSN. However, patients treated with BCG–PSN in combination with antihistamines showed significantly shorter complete relief in time than those treated with antihistamine monotherapy. Since nearly 35% of patients respond poorly to antihistamines (Sanchez-Borges, Caballero-Fonseca & Capriles-Hulett, 2013; Yan et al., 2014), BCG–PSN-combined therapy with the proper dosing may relieve symptoms faster. Although we observed the clinical efficacy of BCG–PSN in CSU, its mediating mechanisms are unknown. Our *in vitro* study of BCG–PSN in RBL-2H3 cells showed a decrease in the β-HEX release rate in the BCG-PSN-treated group without cell toxicity when compared with the control group. In addition, we observed that BCG-PSN enhanced the antihistamine’s inhibitory effect on mast cell degranulation level. Therefore, although BCG-PSN cannot replace antihistamine treatment as a first-line drug, it could be used to accompany antihistamines to relieve symptoms properly. These results suggested that BCG-PSN may be used for adjuvant treatment of CSU.

To further explore the mechanism by which BCG–PSN regulates IgE-sensitized mast cell function, we conducted lncRNA sequencing. According to the sequencing results in rat mast cells, some mRNAs and lncRNAs related to innate immunity and cytokines were aberrantly expressed. ERBB4, also known as HER4, is in a subfamily of epidermal growth factor receptors that was previously reported to activate the MAP kinases MAPK1/ERK2. ERBB4 is highly expressed in Crohn’s colitis and is upregulated by activation of nuclear factor kappa (NF-κB) (Frey et al., 2009). According to the GeneCards Database (http://www.genecards.org/cgi-bin/carddisp.pl?gene=ERBB4&keywords=ERBB4), ERBB4 can bind to transcription factors (TFs) such as signal transducer and activator of transcription 1, AP-1, and NF-κB. Previous studies have demonstrated the role of NFκB in the process of mast cell activation (Kong et al., 2018). NF-κB and other TFs, such as AP-1, mediate IgE-dependent mast cell signaling and induce cytokine and chemokine release (Toniato et al., 2017). As BCG–PSN is an immunomodulator, we supposed that ERBB4 is a positive regulator of BCG–PSN therapy. We observed the enrichment of tyrosine phosphorylation-related GO terms among our differentially expressed genes, including csf2 and ERBB4. These results suggest that ERBB4 mediates IgE-dependent mast cell activation via MAPK/NF-κB signaling. Then, we validated ERBB4 expression by RT-PCR and found that ERBB4 had a lower expression level in BCG–PSN-treated RBL-2H3 cells than in control cells. However, pathway analysis should be performed in future studies to explain these differences in detail.

RSAD2, also known as viperin, is an enzyme in the radical S-adenosylmethionine superfamily (Honarmand Ebrahimi, 2018; Sezin et al., 2017). It is an inhibitory protein against many viruses such as flaviviruses and participates in cell metabolic reprogramming (Honarmand Ebrahimi, 2018). Furthermore, RSAD2 mediates T cell immune responses. These characteristics indicate that RSAD2 might participate in BCG–PSN-mediated immune-related gene regulation. Our RT-PCR results confirmed that RSAD2 was upregulated in BCG–PSN-treated RBL-2H3 cells, perhaps through antiviral activity. The detailed mechanism of this activity needs further exploration. GO annotation was performed to characterize the differentially expressed genes, and our analysis showed that the MAPK cascade and STAT pathway played roles in the BCG–PSN-treated group. Since the MAPK signaling pathway regulates mast cell function via phosphorylation of ERK, JNK, and IκB (Kong et al., 2018), our experimental data provide some useful information on potential targets in CSU. Taken together, our data suggest that BCG–PSN might modulate RBL-2H3 cell degranulation by mediating MAPK signaling and the expression of related genes. Detailed research is warranted to investigate the underlying mechanisms.

## Conclusion

BCG–PSN can decrease the β-HEX release rates and regulate mast cell activation in IgE- sensitized RBL-2H3 cells by mediating immune-related gene expression, including ERBB4. This immunomodulator could act as a potential therapeutic target in mast cell related disease and may be applicable as an adjunctive therapy for CSU patients.

##  Supplemental Information

10.7717/peerj.7404/supp-1 Dataset S1 Raw data of all figure and tables

10.7717/peerj.7404/supp-2 Table S1 The RT-PCR primers used for validation

10.7717/peerj.7404/supp-3 Table S2 The efficacy of different drug-treated group in CSU patients UAS7 values alleviated not less than 50% determined as response. The response analysis was evaluated via chia-quare test.

10.7717/peerj.7404/supp-4 Table S3 Sequencing statistics quality analysis of RBL-2H3 cells treated with or without BCG-PSN Triplicate cell samples were collected. Q30% means the proportion of mass fraction no less than 30% of the total number in the high-quality segment.

10.7717/peerj.7404/supp-5 Table S4 Up and down-regulated differential genes by log2 fold change in RBL-2H3 cells pretreated with or without BCG-PSN

10.7717/peerj.7404/supp-6 Table S5 Up and down-regulated differential genes by log2 fold change in RBL-2H3 cells pretreated with or without BCG-PSN

10.7717/peerj.7404/supp-7 Figure S1 The final predicted lncRNA overview through sequencing statistics (A) LncRNA prediction through three predictive software. Through coding non-coding index (CNCI), coding potential assessing tool (CPAT) and predictor of long non-coding RNAs and mRNAs based on k-mer scheme (PLEK) interactive analysis, 1320 candidate lncRNAs were identified. (B) Subtypes of candidate lncRNA. (C) The proportion of each subtype candidate lncRNA.

## Additional Information and Declarations

### Data Availability

The following information was supplied regarding data availability: The raw measurements are available in Dataset S1.
